# Independent risk factors for death in patients admitted for asthma exacerbation in Taiwan

**DOI:** 10.1038/s41533-020-0164-4

**Published:** 2020-03-19

**Authors:** Yuh-Lih Chang, Hsin-Kuo Ko, Meng-Shui Lu, Chia-Lin Chou, Kang-Cheng Su, Chia-Chen Hsu, Kun-Ta Chou, Tzeng-Ji Chen, Diahn-Warng Perng, Yueh-Ching Chou

**Affiliations:** 1 Department of Pharmacy, Taipei Veterans General Hospital, Taipei, Taiwan; 2 Faculty of Pharmacy, School of Pharmaceutical Sciences, National Yang-Ming University, Taipei, Taiwan; 3 Department and Institute of Pharmacology, National Yang-Ming University, Taipei, Taiwan; 4 Department of Chest Medicine, Taipei Veterans General Hospital, Taipei, Taiwan; 5 School of Medicine, National Yang-Ming University, Taipei, Taiwan; 6 Center of Sleep Medicine, Taipei Veterans General Hospital, Taipei, Taiwan; 7 Department of Family Medicine, Taipei Veterans General Hospital, Taipei, Taiwan; 8 Institute of Hospital and Health Care Administration, National Yang-Ming University, Taipei, Taiwan; 9 School of Pharmacy, Taipei Medical University, Taipei, Taiwan

**Keywords:** Asthma, Prognosis, Epidemiology

## Abstract

The independent risk factors for death in patients admitted for asthma exacerbation have not been thoroughly investigated. This study aimed to investigate these independent risk factors and the relationship between mortality and the prescription patterns of anti-asthmatic medications in patients admitted for asthma exacerbation. Using a nested case–control design, we identified 267 cases (death after asthma admission) and 1035 controls (survival after asthma admission) from the Taiwan National Health Insurance Research Database (NHIRD) from 2001 to 2010. Conditional logistic regressions were used to estimate the odds ratios (ORs) with 95% confidence intervals (CIs). We identified the independent risk factors for death as the comorbidities of pneumonia (aOR 3.82, 95% CI 2.41–6.05), genitourinary disease (aOR 1.75, 95% CI 1.17–2.62), septicemia (aOR 4.26, 95% CI 2.61–6.94), diabetes mellitus (aOR 2.10, 95% CI 1.30–3.38), arrhythmia (aOR 2.00, 95% CI 1.14–3.50), and a history of asthmatic hospitalization (aOR 4.48, 95% CI 2.77–7.25). Moreover, the use of short-acting β_2_-agonist (SABA) and the dosage of oral corticosteroids (OCSs) >70 mg prednisolone during previous hospitalization (all *p* < 0.05) and the dosage of OCSs ≥110 mg prednisolone/month (aOR 2.21, 95% CI 1.08–4.50) during outpatient treatment independently increased the risk of death. The inhaled corticosteroids (ICSs) ≥4 canisters/year (aOR 0.39, 95% CI 0.19–0.78) independently reduced the risk of death. Specific comorbidities, asthma severity, and prescription patterns of SABA, OCSs, and ICSs were independently associated with mortality in patients admitted for asthma exacerbation. These results can be utilized to help physicians identify asthmatic patients who are at a higher mortality risk and to refine the management of the condition.

## Introduction

Asthma exacerbation, characterized by a progressive increase in asthmatic symptoms and a progressive decrease in lung function, accounts for a large proportion of the asthma health-care cost burden.^1^ Patients with severe asthma exacerbation may need emergency department (ED) visits and/or hospitalizations and require changes in asthma treatment.^2–4^ Notably, the frequency of ED visits and hospitalizations for asthma exacerbation is increasing among children, young adults, and the elderly.^5–10^ For patients experiencing asthma exacerbation at ED arrival and on hospital admission, physicians’ knowledge and recognition of mortality risk factors are critical to identifying patients who require additional treatment efforts. However, few studies focus on patients admitted for asthma exacerbation and investigate the independent factors to predict in-hospital mortality in this patient population. Although some epidemiologic predictors (e.g., comorbidities, sex, and age) are associated with mortality in patients hospitalized for asthma exacerbation,^11,12^ the potentially preventable factors associated with mortality have not been thoroughly investigated. Therefore, physicians still face challenges in refining the overall management of asthma and in reducing mortality in patients admitted for asthma exacerbation.

According to health statistics, worldwide asthma mortality has significantly decreased over the past decades; nevertheless, the number of asthmatic patients who die still remains very high.^13–17^ The decrease in asthma mortality may be attributed to the introduction of inhaled corticosteroids (ICSs) for treating asthma over the past 20–30 years.^18^ The high number of deaths from asthma may be explained by a lack of recognition of fatal risk factors in asthmatic patients.^19^ The following risk factors associated with near-fatal asthma and fatal asthma attacks have been reported: (1) comorbidities: a history of psychiatric disease, psychosocial problems, and food allergies; (2) asthma severity: a history of near-fatal asthma attacks requiring intubation and mechanical ventilation, hospitalization or ED visit for asthma in the past year, recently discontinued use of oral corticosteroids (OCSs); (3) a prescription pattern of anti-asthmatic medications: OCS use, not currently using ICS, and overuse of short-acting β_2_ agonist (SABA); and (4) patient compliance: poor adherence to taking asthma medications and/or poor adherence to or lack of a written asthma action plan.^19–25^ Several studies demonstrated that the national database is a useful resource to investigate the risk factors of asthma exacerbation and hospitalization. These studies showed that comorbidity, which existed in older asthma patients, may have contributed to asthma mortality.^11,26,27^ Whether the aforementioned factors can be utilized to independently predict the in-hospital mortality in asthma patients who require ED visits and/or hospitalizations for asthma exacerbation has not been assessed.

Our study aimed to investigate the independent risk factors for death in patients admitted for asthma exacerbation. In particular, we investigated the relationship between mortality and the prescription patterns of anti-asthmatic medications used in both hospitalized and outpatient treatment. We hypothesized that several factors, including comorbidities, asthma severity, the prescription pattern of anti-asthmatic medications, and patient compliance, were independent predictors of death in patients admitted for asthma exacerbation. Moreover, anti-asthmatic medications (e.g., SABA, ICSs, and OCSs) prescribed during hospitalized and outpatient treatment had different impacts on asthma-related deaths. General practitioners and health professionals may be aware of these prognostic factors and refine the asthma management in the community and hospitals to improve patient outcome.

## Results

### Baseline characteristics of the study sample

**Fig. 1:**
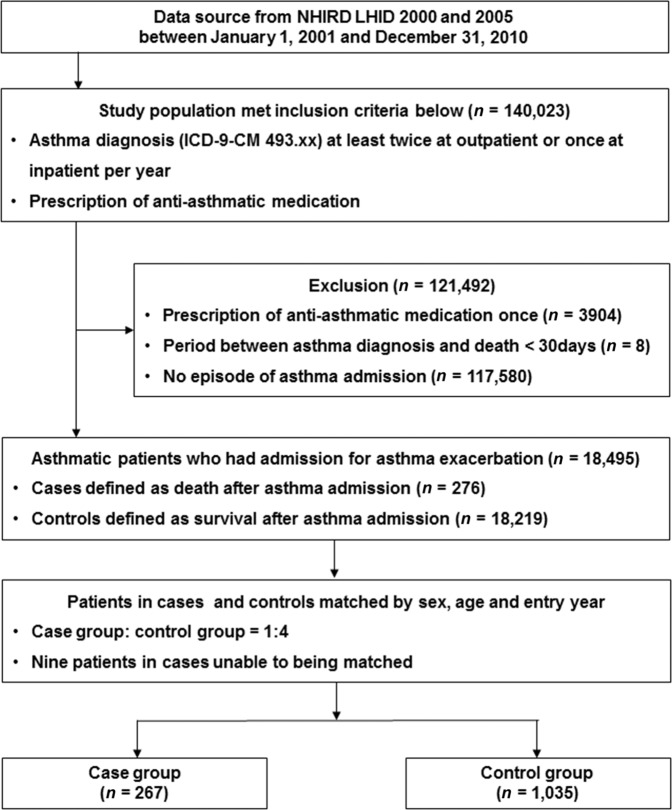
Study flowchart.

In total, 140,023 asthmatic patients who met the inclusion criteria were reviewed from the National Health Insurance Research Database (NHIRD) between 2001 and 2010, and 121,492 asthmatic patients were excluded for the following reasons: (1) they had anti-asthmatic medication administered only once (*n* = 3904); (2) the period between asthma diagnosis and death was <30 days (*n* = 8); and (3) patients were not admitted for asthma exacerbation (*n* = 117,580). Of the 18,495 asthmatic patients who had an ED visit and/or hospital admission for asthma exacerbation, 276 cases of death after asthma admission were identified. The case fatality rates over the 10-year period were 0.2% (276/140,023) for asthmatic patients and 1.5% (276/18,495) for asthmatic patients admitted for asthma exacerbation. Nine patients in the case group could not be matched with the controls. Therefore, 267 eligible cases in the case group and 1035 matched controls (survival after asthma admission) in the control group were analyzed in this study (Fig. 1). The median age of the study sample was 77 years. Most of the patients were in the age range of 61–80 years (45.2%) and most were female (50.7%). The mean duration of follow-up was 3.00 (±2.55) years.

### Factors associated with death after hospitalization for asthma exacerbation

Asthmatic patients who died after admission for asthma exacerbation were significantly associated with the comorbidities of pneumonia (67.8% vs. 21.1%, *p* < 0.001), genitourinary disease (58.8% vs. 23.5%, *p* < 0.001), hypertension (45.3% vs. 30.0%, *p* < 0.001), chronic obstructive pulmonary disease (COPD) (42.3% vs. 23.9%, *p* < 0.001), septicemia (36.7% vs. 6.3%, *p* < 0.001), diabetes mellitus (28.5% vs. 13.9%, *p* < 0.001), heart failure (27.0% vs. 10.7%, *p* < 0.001), ischemic heart disease (23.2% vs. 12.6%, *p* < 0.001), arrhythmia (21.3% vs. 7.9%, *p* < 0.001), gastric ulcer (17.2% vs. 7.7%, *p* < 0.001), and psychiatric disorder (13.1% vs. 5.9%, *p* < 0.001) as compared to the control group (Table 1).

**Table 1:** Baseline patient characteristics in the case and control groups.

	Case (*n* = 267)	Control (*n* = 1035)	*p* values
Age (years)^a^	77 (73–85)	77 (72–84)	0.3758
0–20	0 (0.0)	0 (0.0)	0.9830
21–40	2 (0.8)	8 (0.8)	
41–60	27 (10.1)	106 (10.2)	
61–80	118 (44.2)	470 (45.4)	
>80	120 (44.9)	451 (43.6)	
Sex			
Male	130 (48.7)	512 (49.5)	0.8203
Female	137 (51.3)	523 (50.5)	
Comorbidities^b^			
Pneumonia	181 (67.8)	218 (21.1)	<0.0001
Genitourinary disease	157 (58.8)	243 (23.5)	<0.0001
Hypertension	121 (45.3)	311 (30.0)	<0.0001
COPD	113 (42.3)	247 (23.9)	<0.0001
Septicemia	98 (36.7)	65 (6.3)	<0.0001
Diabetes mellitus	76 (28.5)	144 (13.9)	<0.0001
Heart failure	72 (27.0)	111 (10.7)	<0.0001
Ischemic heart disease	62 (23.2)	130 (12.6)	<0.0001
Arrhythmia	57 (21.3)	82 (7.9)	<0.0001
Gastric ulcer	46 (17.2)	80 (7.7)	<0.0001
Psychiatric disorder	35 (13.1)	61 (5.9)	<0.0001
GERD	10 (3.7)	21 (2.0)	0.1010
Follow-up duration (years)	2.94 ± 2.58	3.02 ± 2.54	0.6340

Data are reported as mean ± S.D. or number (%).

*COPD* chronic obstructive pulmonary disease, *GERD* gastroesophageal reflux disease.

^a^Values are reported as median (interquartile range).

^b^Comorbidities in the year prior to index admission.

Compared with the control group, we found that asthmatic patients who died after admission for asthma exacerbation were significantly associated with a higher percentage of SABA use (92.9% vs. 70.8%, *p* < 0.001) and OCS use (67.4% vs. 50.0%, *p* < 0.001) and a lower percentage of having no anti-asthmatic drug prescriptions (6.0% vs. 14.9%, *p* < 0.001), more frequent asthma-associated health service use (HSU) with ED visits (0.51 ± 1.26 vs. 0.26 ± 1.22, *p* = 0.003), hospitalization (1.09 ± 1.25 vs. 0.2 ± 0.65, *p* < 0.001) and outpatient department (OPD) visits (4.68 ± 7.33 vs. 3.02 ± 5.82, *p* < 0.001). In addition, the case group patients were associated with more frequent ED visits (2.28 ± 3.08 vs. 1.38 ± 1.98, *p* < 0.001) and hospitalization (3.85 ± 2.62 vs. 1.18 ± 2.07, *p* < 0.001) (Table 2).

**Table 2:** Utilization of anti-asthmatic medications and health service use in the case and control groups.

	Case (*n* = 267)	Control (*n* = 1035)	*p* values
Class of anti-asthmatic medication^a^			
SABA	248 (92.9)	733 (70.8)	<0.0001
OCS	180 (67.4)	514 (50.0)	<0.0001
XAN	140 (52.4)	509 (49.2)	0.3428
ICS/LABA fixed dose	38 (14.2)	179 (17.3)	0.2312
LABA alone	2 (0.7)	10 (1.0)	0.7406
ICS alone	16 (6.0)	67 (6.5)	0.7743
LTRA	10 (3.7)	31 (3.0)	0.5315
No prescribed drugs for asthma	16 (6.0)	154 (14.9)	0.0001
Asthma-associated HSU			
ED visit	0.51 ± 1.26	0.26 ± 1.22	0.0034
0	201 (75.3)	872 (84.3)	0.0004
1	38 (14.2)	125 (12.1)	
≥2	28 (10.5)	38 (3.7)	
Hospitalization	1.09 ± 1.25	0.2 ± 0.65	<0.0001
0	106 (39.7)	881 (85.1)	<0.0001
1	87 (32.6)	125 (12.1)	
≥2	74 (27.7)	29 (2.8)	
OPD visit	4.68 ± 7.33	3.02 ± 5.82	0.0007
0	123 (46.1)	526 (50.8)	0.0046
1–2	34 (12.7)	186 (18.0)	
>3	110 (41.2)	323 (31.2)	
HSU 1 year before index date			
ED visit	2.28 ± 3.08	1.38 ± 1.98	0.0002
0	84 (31.5)	466 (45.0)	<0.0001
1–2	86 (32.2)	376 (36.3)	
≥3	97 (36.3)	193 (18.6)	
Hospitalization	3.85 ± 2.62	1.18 ± 2.07	<0.0001
0	10 (3.7)	522 (50.4)	<0.0001
1–2	77 (28.8)	367 (35.5)	
≥3	180 (67.4)	146 (14.1)	
OPD visit	18.59 ± 12.38	20.56 ± 10.90	0.4456
0	18 (6.7)	12 (1.2)	<0.0001
1–12	27 (10.1)	110 (10.6)	
13–24	46 (17.2)	213 (20.6)	
>24	176 (66.0)	700 (67.6)	

Data are reported as mean ± S.D. or number (%).

*COPD* chronic obstructive pulmonary disease, *ED* emergency department, *GERD* gastroesophageal reflux disease, *HSU* health service use, *ICS* inhaled corticosteroid, *LABA* long-acting beta2-agonist, *LTRA* leukotriene receptor antagonist, *OCS* oral corticosteroid, *OPD* outpatient department, *SABA* short-acting beta2-agonist, *XAN* xanthine derivatives.

^a^History of asthma drug use in the year prior to index admission.

**Fig. 2:**
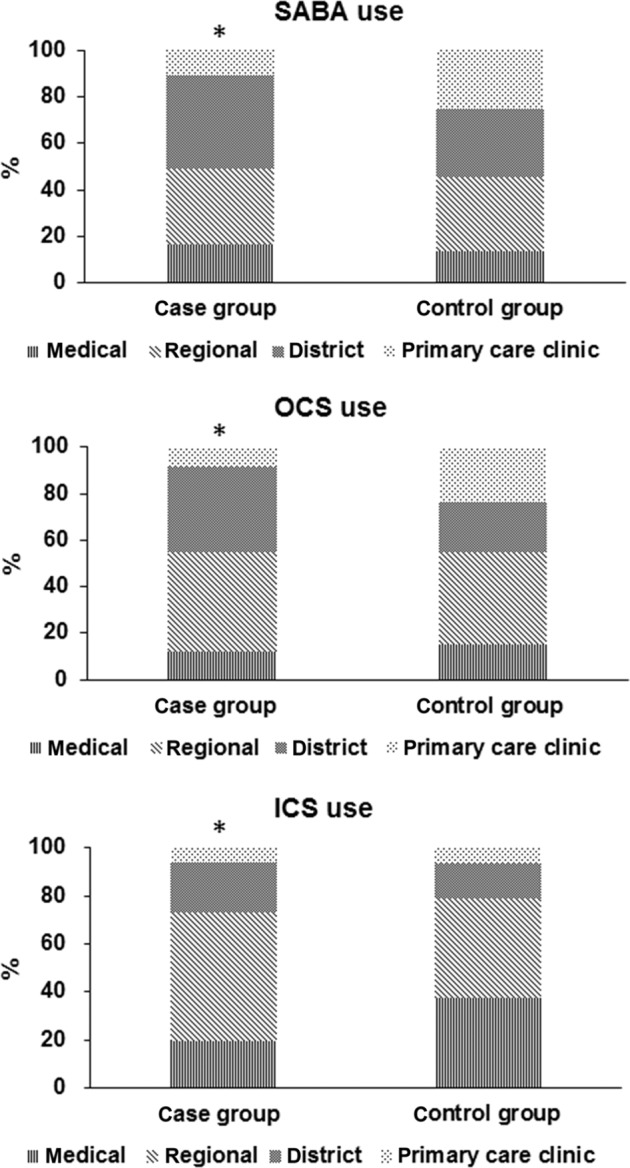
Prescription pattern of anti-asthmatic medication.

The prescription patterns of SABA, OCSs, and ICSs based on hospital levels in the case and control groups were analyzed as shown in Table 3 and Fig. 2. The prescriptions for SABA and OCSs in district hospitals were significantly higher in the case group than in the control group (39.8% vs. 29.5% and 37.0% vs. 21.9%; respectively). Prescriptions for ICSs in medical centers were higher in the control group than in the case group (37.7% vs. 19.2%).

**Table 3:** Prescription patterns of anti-asthmatic medications based on hospital levels.

Prescription	Case (*n* = 267)	Control (*n* = 1035)	*p* values
SABA total	4181	7049	<0.0001
Medical center	682 (16.3)	976 (13.8)	
Regional hospital	1379 (33.0)	2217 (31.5)	
District hospital	1662 (39.8)	2082 (29.5)	
Clinic	458 (11.0)	1774 (25.2)	
OCS total	1769	2821	<0.0001
Medical center	214 (12.1)	418 (14.8)	
Regional hospital	750 (42.4)	1119 (39.7)	
District hospital	655 (37.0)	618 (21.9)	
Clinic	150 (8.5)	666 (23.6)	
ICS total^a^	286	1326	<0.0001
Medical center	55 (19.2)	500 (37.7)	
Regional hospital	153 (53.5)	548 (41.3)	
District hospital	60 (21.0)	191 (14.4)	
Clinic	18 (6.3)	87 (6.6)	

Data were reported as numbers (%).

*SABA* short-acting β_2_-agonist, *OCS* oral corticosteroid, *ICS* inhaled corticosteroid.

^a^Prescription numbers were the sum of ICS monotherapy and the fixed-drug combination of ICS and the long-acting β_2_-agonist.

### Independent risk factors for death in patients admitted for asthma exacerbation

**Fig. 3:**
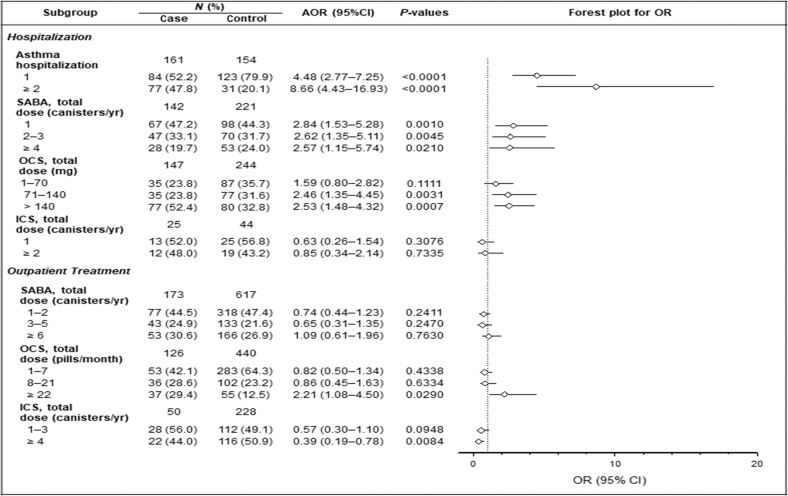
Forest plot for subgroup analyses of the risk of death.

In the multivariable logistic regression analysis, the comorbidities of pneumonia [adjusted odds ratio (aOR) 3.82, 95% confidence interval (CI) 2.41–6.05; *p* < 0.001], genitourinary disease (aOR 1.75, 95% CI 1.17–2.62, *p* = 0.006), septicemia (aOR 4.26, 95% CI 2.61–6.94; *p* < 0.001), diabetes mellitus (aOR 2.10, 95% CI 1.30–3.38, *p* = 0.003), and arrhythmia (aOR 2.00, 95% CI 1.14–3.50, *p* = 0.016) remained as the independent risk factors for death. In asthmatic patients hospitalized once or twice or more for asthma exacerbation in the preceding year, the risk of death increased 4.48- and 8.66-fold, respectively (aOR 4.48, 95% CI 2.77–7.25, *p* < 0.001 and aOR 8.66, 95% CI 4.43–16.93, *p* < 0.001) (Table 4 and Fig. 3).

**Table 4:** Risk factors for death in asthmatic patients hospitalized for asthma exacerbation as analyzed by bivariable and multivariable logistic regression.

	Crude OR (95% CI)	*p* values	AOR^a^ (95% CI)	*p* values
Comorbidities				
Pneumonia	9.14 (6.48–12.90)	<0.0001	3.82 (2.41–6.05)	<0.0001
Genitourinary disease	4.67 (3.48–6.27)	<0.0001	1.75 (1.17–2.62)	0.0064
Hypertension	1.94 (1.47–2.57)	<0.0001	0.90 (0.59–1.38)	0.6396
COPD	2.42 (1.81–3.24)	<0.0001	0.79 (0.50–1.26)	0.3202
Septicemia	8.47 (5.82–12.33)	<0.0001	4.26 (2.61–6.94)	<0.0001
Diabetes mellitus	2.63 (1.89–3.66)	<0.0001	2.10 (1.30–3.38)	0.0025
Heart failure	3.13 (2.21–4.44)	<0.0001	1.23 (0.73–2.08)	0.4410
Ischemic heart disease	2.09 (1.48–2.94)	<0.0001	1.09 (0.66–1.81)	0.7301
Arrhythmia	3.20 (2.17–4.73)	<0.0001	2.00 (1.14–3.50)	0.0157
Gastric ulcer	2.58 (1.73–3.85)	<0.0001	1.07 (0.59–1.96)	0.8155
Psychiatric disorder	2.38 (1.83–3.70)	<0.0001	1.00 (0.54–1.85)	0.9866
GERD	1.91 (0.90–4.05)	0.0935	0.86 (0.30–2.50)	0.7868
Asthma ED visit				
1	1.36 (0.91–2.03)	0.1050	0.57 (0.32–1.02)	0.2516
≥2	3.24 (1.94–5.41)	0.0002	0.88 (0.40–1.92)	0.6853
Asthma hospitalization				
1	5.98 (4.10–8.73)	<0.0001	4.48 (2.77–7.25)	<0.0001
≥2	17.83 (10.83–29.34)	<0.0001	8.66 (4.43–16.93)	<0.0001
Asthma OPD visit				
1–3	0.91 (0.62–1.33)	0.6221	0.84 (0.49–1.44)	0.5313
≥4	1.48 (1.09–2.02)	0.0131	1.10 (0.68–1.79)	0.6930

^a^Adjusted for age, sex, HSU, asthma medication, comorbidities, OCSs, and SABA dosage.

During hospitalized treatment in the preceding year, use of SABA and a dosage of OCS > 70 mg prednisolone independently increased the risk of death (all *P* < 0.05). These findings indicated a previous ED visit and/or hospital admission caused by a serious asthma exacerbation requiring SABA use and a higher dosage of OCS use as an important predictor for asthma-related death (Fig. 3). During outpatient treatment, a dosage of OCS ≥ 22-pill (110 mg) prednisolone/month in the preceding year independently increased the risk of death (aOR 2.21, 95% CI 1.08–4.50, *p* = 0.029), and ICS ≥ 4 canisters per year in the preceding year independently reduced the risk of death (aOR 0.39, 95% CI 0.19–0.78, *p* = 0.008).

## Discussion

For patients with exacerbated asthma upon ED arrival and on hospital admission, the independent risk factors for death can help physicians identify those at a higher risk for death and those who need more specialized attention. In this study, we reported independent mortality risk factors in patients admitted for asthma exacerbation as (1) comorbidities including pneumonia, genitourinary disease, septicemia, diabetes mellitus, and arrhythmia; (2) asthma severity with a history of asthma-associated hospitalization and use of SABA and a dosage of OCS use >70 mg prednisolone during hospitalized treatment in the preceding year; and (3) a prescription pattern with dosage of OCS use ≥110 mg prednisolone/month during outpatient treatment in the preceding year. Notably, the prescription for ICS dosage ≥4 canisters/year during outpatient treatment in the preceding year was independently associated with the reduced mortality risk. Meanwhile, we found that the prescription for SABA and OCSs was more frequent in district hospitals, and the prescription for ICSs was more frequent in medical centers as shown by a comparison between the case and the control groups. Physicians may use the results of our study to refine asthma management and improve the outcomes in patients admitted for asthma exacerbation.

In this study, we reported that the case fatality rate over the 10-year period was 1.49% (276/18,495) in hospitalized asthmatic patients. Watson et al. utilized a national United Kingdom database analysis of the asthma burden in the inpatient hospital setting and reported the mortality rate of 0.43% over the 5-year study period.^11^ In Watson’s study, the percentage of hospitalized asthmatic patients aged <44 years was 73%; however, the percentage of asthmatic patients aged <60 years was only 10% in our study. An older age in the study population may have contributed to a higher rate of in-hospital mortality in our study as compared to previous studies.^11,12^ The study population in this study was relatively old. More than 90% of our population was aged >60 years. To ensure that our population represented the asthma population, we used the same inclusion criteria to include patients who died after acute asthma exacerbation from 2008 to 2011, and we calculated the crude death rate. The results showed that the crude death rate was 0.027‰, 0.025‰, 0.029‰, and 0.031‰, respectively, from 2008 to 2011. We found that these patients’ death rate was comparable to the asthma-related death data released by the Taiwan Ministry of Health and Welfare (0.032‰, 0.026‰, 0.025‰, and 0.027‰, respectively).^28^ Therefore, we believe that the results from our study population represent the population affected by asthma-related death.

Watson et al. analyzed a national United Kingdom database and reported that comorbidities of respiratory infection, cardiovascular disease, and diabetes are common in older asthma patients and may contribute to mortality after an asthma-related admission.^11^ Indeed, we identified that comorbidity conditions, including pneumonia, genitourinary disease, septicemia, diabetes, and arrhythmia, were independently associated with the increased risk of death in patients admitted for asthma exacerbation. Therefore, we suggest that an understanding of epidemiological risk factors and comorbidities is important for physicians so that they can identify asthmatic patients who are at a higher risk of death at ED visit and asthma admission.

Factors associated with asthma severity, including a history of intubation and mechanical ventilation support, hospitalization or ED visits for asthma, and current use or recent discontinuation of OCS, have been associated with near-fatal and fatal asthma in previous studies.^22,24,25^ Guite et al. investigated the risk factors for asthma-related death occurring within 3 years of discharge from hospital admission for asthma.^24^ The authors reported the independent risk factors associated with death from asthma as a history of clinically severe asthma, chest pain, biochemical or hematological abnormalities at admission, prescription for ipratropium bromide, and failure to prescribe ICSs on discharge. Kang et al. also reported that a history of corticosteroid bursts was significantly associated with asthma exacerbation. A history of corticosteroid bursts also was a risk factor for future exacerbation among all severities.^26^ Here we demonstrated that the indicators of asthma severity, including a history of asthma-associated hospitalization, use of SABA, and a dosage of OCS use >70 mg prednisolone during hospitalized treatment in the preceding year, were independently associated with fatal asthma. These findings indicate that asthma severity is the important determinant associated with near-fatal/fatal asthma and asthma-related mortality after discharge of an asthma admission.

Chou et al. analyzed prescription patterns for managing asthma in newly diagnosed patients in Taiwan.^29^ The authors reported an increased use in the fixed-dose combination of ICS/long-acting β2-agonist (LABA) in Taiwan (from 3.6% in 2002 to 28.8% in 2010) and an increased trend in guideline adherence with the concomitant use of LABA and ICSs in academic medical centers and asthma specialists. In this study, we further confirmed that the guideline adherence with ICS/LABA or ICS ≥4 canisters/year during outpatient treatment significantly reduced the mortality risk in patients admitted for asthma exacerbation. It also implies that strong patient compliance with the ICS maintaining treatment for asthma control is associated with reduced mortality in patients admitted for asthma exacerbation. Chou’s study reported a reduced use of OCSs and SABA for asthma management after 2005 in Taiwan.^29^ However, the use of SABA and a high dosage of OCSs during hospitalized treatment were still independently associated with the increased risk of death in patients admitted for asthma exacerbation. We observed that prescription patterns based on hospital levels showed a higher percentage of SABA and OCS use in district hospitals in the case group as compared to that in the controls. The use of ICSs in medical centers was higher in the control group than in the case group. We suggested that analyzing prescription patterns based on hospital levels may help institutions define guideline adherence and improve the quality of health care in asthma management.

In order to reduce asthma-related mortality, we need better implementation and management strategies. The UK National Review of Asthma Deaths (NRAD), which reported that following the treatment guideline recommendation could prevent nearly half of asthma-related deaths,^30^ revealed the importance of standard treatment in clinical practice. Based on the NRAD findings, six areas for improving asthma care: prevention of attacks and avoidable deaths, including systems for early, accurate diagnosis of asthma; providing care; identification of risk; implementation of guidelines; patient education and provision of personal asthma action plans; and improved quality of completion of medical certificates specifying the cause of death, need to be fully considered. Analysis of Australian asthma mortality showed that >70% of asthma deaths have preventable or modifiable factors.^31^ The authors suggested that the interventions should be done at the patient level, at the health professional level, and at the policy level. Patients and their caregivers should improve their knowledge of asthma, and self-management education is needed. Health professionals should ensure that they provide patients with enough education and advice on a treatment plan. At the policy level, the managing authority should ensure the appropriateness of the national guidelines and should highlight the solutions to meet the needs of clinical circumstances. For high-risk patients, such as those who visit the ED, asthma education and an asthma action plan should be required as part of a national policy.

Menzies-Gow et al. offered six core principles: timely diagnosis, referral, support of patients by providing relevant information about asthma and personalized education, treatment not reliant on OCSs, and access to consistent quality care, to address the unmet need in treating severe asthma and to deliver improvements in asthma care.^32^ These strategies, based on the latest understanding of the disease, should be used to benchmark current service provisions. The results of this study will help health-care professionals to identify those who are at a higher risk of dying and those who need more specialized attention. Collectively, we urge policy makers and those responsible for the delivery of asthma care to apply our results to modify the care plans to improve the quality of care and reduce the rate of asthma mortality.

Our study has some limitations. First, we recorded and assessed mortality risk factors in the year prior to the index date for all cases and controls. Factors prior to 1 year before the index date and during index admission were not recorded and analyzed. The influence of these factors on asthma-related death is unknown. Second, records for lung function, asthma symptom scores, and patient adherence to anti-asthmatic medications were not available in the National Institutes of Health database. Therefore, asthma severity was indirectly assessed by the prescription pattern of anti-asthmatic medications (e.g., use of SABA and OCSs). The influence of patient compliance on the risk of death in asthmatic patients hospitalized for asthma exacerbation remains unclear. Third, our study investigated the risk factors for death in asthma patients admitted for asthma exacerbation. The results of our study were unable to demonstrate the occurrence of premature death due to asthma. The report of global, regional, and national disability-adjusted life years might be more appropriate to reflect premature death resulting from asthma.^33^

In patients admitted for asthma exacerbation, we identified the independent risk factors for death as (1) the comorbidities of pneumonia, genitourinary disease, septicemia, diabetes, and arrhythmia, (2) asthma severity with a history of asthma-associated hospitalization and use of SABA and dosage of OCS >70 mg prednisolone during hospitalized treatment in the preceding year, and (3) prescription patterns with a high dosage of OCS ≥110 mg prednisolone/month during outpatient treatment. Notably, the use of ICS ≥4 canisters/year was independently associated with a reduced mortality risk. In addition, analyzing prescription patterns of anti-asthmatic medications based on hospital levels may help institutions define the guideline adherence and improve the quality of health care in asthma management. These independent factors reported in our study can be utilized to help physicians to identify asthmatic patients who are at a higher mortality risk and to refine asthma management.

## Methods

### Ethics statement

We consulted with the Institutional Review Board (IRB) of Taipei Veterans General Hospital, Taipei, Taiwan whose members waived the requirement for informed consent for our retrospective observational study. The study was registered at the IRB of Taipei Veterans General Hospital, Taipei, Taiwan (IRB 2015-07-001BC), and the approval date was July 9, 2015.

### Data source

We obtained data from the Taiwan NHIRD from January 1, 2001 through December 31, 2011. The dataset contains medical records of approximately 23 million insured enrollees, representing 99% of the registered people in Taiwan. The NHIRD medical records included demographic information, pharmaceutical records, procedures undertaken, major patient outcomes, and diagnosis. The diagnosis codes were encoded using the International Classification of Disease, Ninth Reversion, Clinical Modification (ICD-9-CM). The accuracy of the insurance claims data in Taiwan was validated, with 92.4% accuracy for patients with ≥1 hospitalization in a year.^34^

### Study sample

The retrospective study data, collected from the Longitudinal Health Insurance Database for the years 2000 and 2005, were based on original claim data. The data set for each of these years contained one million randomly sampled patients. The asthma cohort inclusion criteria consisted of patients who had been diagnosed as asthmatic (ICD-9-CM 493.xx) at least twice as outpatients or once as inpatients per year and who were given any of the following anti-asthmatic medications after a first-diagnosis date between January 1, 2001 and December 31, 2010: ICS, OCS, SABA, LABA, leukotriene receptor antagonist (LTRA), xanthium derivatives (XAN), and anti-immunoglobulin E (anti-IgE) therapy. The entry date used for the asthma cohort was the patient’s first-diagnosis date. The period of each individual’s follow-up was from the entry date up to (1) any occurrence of hospital admission because of asthma exacerbation, (2) the patient’s death after hospital admission for asthma exacerbation, (3) the lapse of the patient’s health insurance registration, and (4) the end of the study (December 31, 2011). Exclusion criteria included (1) anti-asthmatic medication was administered only once after asthma diagnosis; (2) the period between the first-diagnosis date of asthma and death was <30 days; and (3) patients were not admitted for asthma. Asthma admission was defined as patients who had an ED visit and/or were hospitalized for asthma exacerbation and where the primary ICD code 493.xx (asthma diagnosis) was registered at the same time. We identified eligible cases for study inclusion as all individuals in the asthma cohort who had experienced asthma admission and fatal exacerbation within the study period, and we defined the death date as the index date. The controls were defined as individuals in the asthma cohort who had experienced asthma exacerbation and were alive on the index date. Four controls, randomly selected for each case, matched by sex, age (±2), and entry year. The study sample was divided into case group (death after hospitalization for asthma exacerbation) and control group (survival after hospitalization for asthma exacerbation) (Fig. 1).

### Assessment of risk factors for death in patients admitted for asthma exacerbation

This study examined several variables potentially associated with fatal asthma, including demographics, HSU, comorbidities, anti-asthmatic medications, and drug adherence in the year prior to the index date for all cases and controls. Patient comorbidities diagnosis data (according to ICD-9-CM codes) were collected from the year before the entry date. These included pneumonia, genitourinary disease, hypertension, COPD, diabetes mellitus, heart failure, ischemic heart disease, arrhythmia, gastric ulcer, psychiatric disorder, and gastroesophageal reflux disease. Anti-asthmatic medications included in this analysis were LABA, SABA, ICS, OCS, LTRA, XAN, and Anti-IgE, which are the recommended asthma management medications listed in the guidelines of the Global Initiative for Asthma.^35^ We also evaluated existing prescription patterns of anti-asthmatic medications during hospitalized and outpatient treatment. In addition, we evaluated how these patterns varied among different medical institutions, such as medical centers, regional and district hospitals, and outpatient clinics. We defined medication users as patients whose cumulative dispensed dose of controller medications was for >30 days in the year preceding the index date. The dose of SABA was converted to canisters (200-puff) of salbutamol (100 mcg/puff) or the equivalent, and OCS was converted to prednisolone (5 mg/pill) equivalent fillings.

### Statistical analysis

We analyzed the data using the Microsoft SQL Server 2008 and SAS version 9.4 software. Descriptive statistics for both the case and control groups included demographic characteristics, asthma-associated health-care utilization, distribution of comorbidities, and anti-asthmatic medications. Statistics were compared using *χ*^2^ test for categorical variables and *t* tests for continuous variables; results were considered statistically significant at *p* < 0.05. All *p* values were two sided. Conditional logistic regression was used to measure OR and 95% CIs of each variable on asthma mortality, with adjustment for age, sex, HSU, comorbidities, anti-asthmatic medications, and SABA and OCS dosage.

### Reporting summary

Further information on research design is available in the Nature Research Reporting Summary linked to this article.

## Supplementary information

Reporting Summary

## Data Availability

The data that support the findings of this study are available on request from the corresponding author. The data are not publicly available because the use of the National Health Insurance Research Database is limited to research purposes only.
